# Editorial: Structural and mechanistic determinants of endurance performance

**DOI:** 10.3389/fphys.2022.1035583

**Published:** 2022-10-14

**Authors:** Fábio Juner Lanferdini, Fernando Diefenthaeler, Luca Paolo Ardigò, Leonardo Alexandre Peyré-Tartaruga, Johnny Padulo

**Affiliations:** ^1^ Laboratório de Biomecânica, Universidade Federal de Santa Maria, Santa Maria, Brazil; ^2^ Laboratório de Biomecânica, Universidade Federal de Santa Catarina, Florianópolis, Brazil; ^3^ Department of Teacher Education, NLA University College, Oslo, Norway; ^4^ Department of Neurosciences, Biomedicine and Movement Sciences School of Exercise and Sport Science, University of Verona, Verona, Italy; ^5^ Laboratório de Pesquisa do Exercício, Universidade Federal do Rio Grande do Sul, Porto Alegre, Brazil; ^6^ Department of Biomedical Sciences for Health, Università degli Studi di Milano, Milan, Italy

**Keywords:** determinants, predictors, endurance sports performance, anthropometry, biomechanical, neuromuscular, physiological

Endurance performance is a multifactorial phenomenon. Physiologically, endurance performance depends on high maximal oxygen uptake, anaerobic thresholds, and movement economy. The precise mechanisms mediating these factors with respect to metabolic/muscular efficiency (mitochondrial amount, muscle capillarization, and others) are still unclear. Regarding biomechanics, the high economy stems from executing ideal mechanical patterns that involve the application of forces with the appropriate magnitude, direction, and timing while avoiding non-productive movements as an uncoordinated gait to optimize the transmission efficiency (Peyré-Tartaruga and Coertjens, 2018). In addition, biomechanical factors, such as muscle size, muscle architecture and quality, and tendon mechanical properties, can also help to determine long-term sports endurance performance. Endurance sports performance can also be understood in terms of muscle recruitment (e.g., muscle activation, or neuromuscular economy) during a specific motor task to be performed in that sport. Anthropometric factors, such as height, body mass, percentage of lean mass, and length of the upper or lower limbs, can also prove to be crucial for understanding which variables can significantly determine endurance sports performance.

## Endurance determinants

The present manuscript reports interesting information on the complexity of the factors affecting endurance performance. Despite this complexity, most of the studies included in this editorial investigated the effects of physiological variables on endurance performance. Indeed, Figueiredo et al. demonstrated the value of peak running velocity and critical speed in determining 5-km running performance, suggesting that the well-structured and periodized training program should take these variables into consideration to improve the 5-km performance of recreational runners. Moreover, foot strike patterns seem to change across running speeds according to the foot strike index. Ekizos et al. observed a similar foot strike index with increased speeds in most runners (particularly in rearfoot strikers). However, some mid-forefoot strikers decrease the foot strike index with increasing speed. This could have implications for the metabolic energy consumption of mid-forefoot striker runners, typically measured at low speeds for the assessment of running economy. The higher foot strike index in the mid-forefoot striker runners results in distinct distributions of muscular output in the lower extremities compared with rearfoot strikers (i.e., higher moments at the ankle and lower moments at the knee joint for mid-forefoot striker runners). Additionally, Fong and Powell showed that greater breast support is associated with improved oxygen consumption (absolute and relative) and running economy in women runners. The breast support effect on oxygen consumption and running economy is influenced by breast size, with larger breasted athletes obtaining greater improvements in running performance than smaller breasted women. The use of face masks during the incremental running test affected physical and cognitive performance and maximal oxygen uptake in Slimani et al. study, suggesting the importance of avoiding cloth face mask use during maximal aerobic tests.

Otherwise, Alejo et al. reported that physiological endurance cycling indicators (e.g., maximum oxygen uptake, peak power output and respiratory compensation point) and 8-min time-trial performance are greater in professional and under-23 years cyclists than in junior cyclists. Furthermore, professional cyclists presented a higher ventilatory threshold compared with under-23 and junior cyclists. Some differences were also found in anthropometric parameters, with professional cyclists showing a lower relative fat mass and higher muscle mass than under-23 and junior cyclists. Similarly, no consistent differences were found between age categories for strength/power indicators. However, the results of Kominami et al. showed that the respiratory compensation point and ventilatory threshold are poor indicators of lactate buffering capacity without robust effects with age during exercise. Exercise tolerance decreases with age. Furthermore, mixed-reality sports can be potential approaches for endurance performance. Westmattelmann et al. investigated the influence of different power parameters, body mass and height on Virtual cycling Tour de France 2020 performance. The results showed that relative power output explains variance in performance. In addition, the authors explained that body mass and height can explain the results in only a few stages of the Virtual Tour de France, and lower body mass in particular determines race success in the mountain stages. Understanding human endurance performance is a complex task that increasingly demands from researchers, coaches and athletes knowledge of the multiple components involved in assessments and training programs. In this manner, endurance performance is likely to defy the types of easy explanations sought by scientific reductionism and remain an important puzzle for those interested in biomechanics and physiological integration, among other aspects to be explored in the future (Joyner and Coyle, 2008).

Contrary to the findings for endurance indicators, intermittent sports (e.g., taekwondo) involve high-intensity movements interspersed by periods of low intensity. Sant’Ana et al. observed that the internal load of the roundhouse kick corresponds to the anaerobic threshold in taekwondo athletes and can be considered in the training prescription. In taekwondo, when planning a training program, it is necessary to consider the specific demands of the sport and its intermittent characteristics. Finally, Borghi-Silva et al. found that unloading the respiratory musculature with proportionally assisted ventilation accelerates haemodynamic and muscle oxygenation recovery. These beneficial effects improve tolerance to repeated (interval) exercise in patients with heart failure with a reduced left ventricular ejection fraction.

Therefore, based on the studies submitted for this editorial, we integrated physiological performance determinants for metabolic implications, especially on cycling and running endurance sports. Based on the present editorial, key physiological determinants may serve as targets for training strategies in sports such as cycling and running to optimize endurance performance.

## Looking to the future

Some physiological variables (e.g., maximum oxygen uptake, ventilatory threshold and movement economy) discussed in the present manuscript particularly influence the determinants of endurance performance. The present article presented some exciting new factors affecting endurance performance. Determinants of endurance sports performance have been established over the last few decades, particularly the effect of physiological factors. However, few studies have aimed to understand the determinants of sports performance with integrative use in the different areas proposed by this editorial. However, multicomponent characteristics can be directly related to cycling endurance performance by highlighting the importance of anthropometric characteristics and cycling training potential over classically held traditional variables in relation to the limits of human performance. Therefore, it is necessary to investigate multiple components in more ecological tests for the evaluation of endurance performance. Finally, other components (e.g., integrating anthropometry, biomechanics, and physiology) may present new evidence to determine endurance performance and should be investigated in ecological conditions (Batterson et al., 2020; Kordi et al., 2020; Bohm et al., 2021). Furthermore, we hope that the present Research Topic will stimulate more researchers to pursue answers regarding the determinants of endurance performance by integrating anthropometric, physiological and biomechanical analyses, aiming to provide more solid research knowledge and subsequent practical application.
